# Activity and Biocompatibility Evaluation of Enzybiotic Compositions Formulated with *Azotobacter vinelandii* Alginate for Topical Use

**DOI:** 10.3390/ijms27093856

**Published:** 2026-04-27

**Authors:** Arina A. Klimova, Ekaterina A. Trusova, Elizaveta A. Akoulina, Nataliia P. Antonova, Andrei A. Dudun, Michail Yu. Orlov, Marina Yu. Kochevalina, Vera V. Voinova, Anton P. Bonartsev, Daria V. Vasina

**Affiliations:** 1 N.F. Gamaleya National Research Centre for Epidemiology and Microbiology, Ministry of Health of the Russian Federation, 123098 Moscow, Russia; arina.klimova10012001@gmail.com (A.A.K.); northernnatalia@gmail.com (N.P.A.); dudunandrey@mail.ru (A.A.D.); 2 Faculty of Biology, MSU-BIT Shenzhen University, Shenzhen 518115, China; trusova.cat2017@yandex.ru (E.A.T.); akoulinaliza@gmail.com (E.A.A.); 3 Faculty of Biology, M.V. Lomonosov Moscow State University, 119234 Moscow, Russia; o.mike.u@live.ru (M.Y.O.); mkochevalina@gmail.com (M.Y.K.); veravoinova@mail.ru (V.V.V.); ant_bonar@mail.ru (A.P.B.); 4 Research Center of Biotechnology of the Russian Academy of Sciences, 119071 Moscow, Russia

**Keywords:** recombinant endolysin, bacterial alginate, topical gels, hydrogel, safety, wound material

## Abstract

Due to their availability and environmental friendliness, alginate polymers are widely used in pharmaceuticals and cosmetics. The most common type of alginate is derived from seaweed and is used to develop topical dosage forms, among other things. However, variability in the seaweed material can lead to instability in the physicochemical parameters. Biotechnologically produced alginate minimizes this drawback through controlled synthesis. However, unlike algal alginates, the safety profile of such polymers has not been well studied. When developing dosage forms intended for wound surfaces, safety is of primary importance. In this study, we developed enzybiotic compositions based on bacterial alginate as an excipient and a novel recombinant modified endolysin, LysSi3-LK, as an antibacterial agent, and assessed their antibacterial properties and safety profile. The study included an *in vitro* evaluation of the activity spectrum, as well as the cytotoxicity and biocompatibility, of gel and hydrogel compositions. It was demonstrated that bacterial alginate is acceptable for the encapsulation of endolysin. It exhibited medium cytotoxic effects on the HaCaT cells, which were significantly reduced by the LysSi3-LK addition. The migration of cells was diminished following exposure to the gel and hydrogel formulations. However, an improvement in biocompatibility was observed in the cell proliferation assay.

## 1. Introduction

Antimicrobials containing bacteriolytic enzymes (lysins) are being actively developed as a new class of alternative drugs. These enzymes are effective against antibiotic-resistant bacteria due to their action on a highly conserved bacterial cell wall substrate (peptidoglycan), and have a low potential for resistance formation [1]. In this regard, enzybiotics are considered as one of the promising strategies for combating multidrug-resistant Gram-positive and Gram-negative pathogens [1,2,3].

Nowadays, enzybiotics are primarily considered a topical therapy option in the form of gels [4], ointments [5], wound dressings [6,7], drops [8], and sprays [2]. However, it is known that lysins, especially genetically modified ones that target Gram-negative bacteria, can be cytotoxic. Non-selective antimicrobial peptides in their structure can disrupt the integrity of eukaryotic membranes [9,10]. Although the topical route of administration potentially reduces the risk of systemic side effects, the dosage form comes into contact with damaged skin, which may increase the risk of sensitization and immunological effects after repeated applications. Few results of preclinical safety studies of individual endolysins in topical formulations have been described. A gel containing the modified enzyme LysECD7-SMAP, which is active against Gram-negative bacteria, was shown to have no systemic bioavailability or toxic effects, according to the results of general toxicity, local tolerability, and immunotoxicity studies following single and repeated applications [11]. A study of the immunogenicity of the antistaphylococcal chimeric endolysin ClyS in a petrolatum-based ointment over eight weeks of application showed a low titer of specific antibodies that lacked neutralizing activity [12]. Dosage forms containing the phage-derived antistaphylococcal enzyme Staphefekt are available for practical use as skin care products and have demonstrated hypoallergenicity and skin compatibility [13,14]. These results confirm the feasibility of developing safe, topical forms of endolysins.

The practical application of lysins depends both on their bacteriolytic activity and effective delivery to the site of infection. Their use in the treatment of topical wound infections is limited by the instability of protein molecules upon application to the skin surface, as well as by the need to deliver and maintain the enzyme at an effective concentration in its active form [15]. In addition, the active molecule may be mechanically removed together with wound exudate during the treatment. Therefore, the selection of an appropriate dosage form and delivery system is a key issue for the topical application of lysins.

A number of enzybiotics delivery strategies have already been explored to overcome various biological barriers that hinder the transport of endolysins to the site of infection, including liposomes, nanoparticles and PEGylated systems. Studies on targeted endolysin delivery clearly demonstrate the efficiency of such approaches. E.g., encapsulation in liposomes does not impair the antibacterial activity of endolysins and, in some cases, even broadens their activity spectrum by helping them to overcome the outer membrane of Gram-negative bacteria [16]. PEGylated liposomes have been shown to improve transport across biological barriers [17]. Chitosan nanoparticles have also been used to enhance bioavailability and mucoadhesion [18]. 

By contrast, the wound surface is not characterized by the presence of specific barriers, but instead requires maintenance of an effective local concentration, as well as high biocompatibility and wound-healing properties of active components and vehicle compounds. In this context the use of gels and hydrogels appears more practical and, as was previously shown, supports the effectiveness of this approach. Lysin ClyC loaded in the alginate hydrogel demonstrated sustained release, preservation of enzymatic activity and reduction in bacterial burden *in vivo* [19]. A thermosensitive Poloxamer-based hydrogel containing LysP53 was active against *Acinetobacter baumanii* [6]. 

One can conclude that for bacteriolytic enzymes, the optimal vehicle agents include polysaccharide polymers that are compatible with the protein nature of the active ingredient, as well as biocompatible and safe for the environment [20]. In particular, alginates have a high capacity for water absorption and are readily available [21,22]. Due to their polyanionic nature, alginates have several advantages for delivering cationic endolysins [23]. The negatively charged carboxyl groups form bonds with the positively charged enzyme sites, which is especially important for controlled release under changing pH conditions of the wound environment. The hydroxyl groups of the monosaccharide units create a hydrophilic environment that helps maintain the protein’s native conformation.

Sodium alginate, which is derived from natural materials such as brown seaweed, is currently approved for drug development. It is widely used in research as a biopolymer carrier for the delivery and controlled release of biologically active compounds and proteins [24,25,26]. It is important to highlight that the production and physicochemical characteristics of algal alginate primarily depend on the season and growing conditions of the algae. Although some companies (e.g., FMC Corporation, Novamatrix) control the composition of alginates isolated from algae (e.g., by collecting algae from different species and regions), the natural characteristics of alginate are difficult to control. Thus, technology for collecting and processing algal alginates limits production of the excipient with specified characteristics for biomedical use, regardless of environmental conditions. This makes it difficult to rely on stable material production when even small changes in polymer composition can affect the controlled release.

Bacteria of the Azotobacter and Pseudomonas genera are an alternative source of alginate [27]. In the case of bacterial alginates, a biotechnological approach to the synthesis of biopolymers is possible, ensuring the process is both biologically safe and effective. Modifying the cultivation conditions can influence the composition and ratio of monomers, as well as the molecular weight of bacterial alginate, to produce polymers with the desired properties for specific applications [28]. Furthermore, bacterial alginate, in contrast to algal alginate, can undergo acetylation during biosynthesis, a process that also impacts the characteristics of the resulting polymer. While the extent of this influence remains unclear, it is believed that acetylation makes calcium hydrogels softer than non-acetylated polymers [29]. During isolation and purification, bacterial alginates separate into two fractions: free, low-molecular-weight alginate (below 150 kDa), which is incapable of forming stable hydrogels, and high-molecular-weight capsular alginate (above 200 kDa). Thus, the purification process produces alginate with a smaller molecular weight range [30].

Although drug development using lysins active against a range of pathogens is ongoing, relatively few hydrogel studies have addressed Gram-negative targets, despite the importance of this direction. Currently, there are few studies describing the safety and biocompatibility of these protein-polysaccharide compositions based on bacterial alginates [31,32]. Despite the data indicating the good tolerability of certain endolysin-containing topical agents and the general information regarding the biocompatibility of alginate materials, a methodologically important gap remains. Only a limited number of studies have comprehensively assessed the safety of the alginate-endolysin combination, taking into account cytotoxicity, local tolerability, and potential immunological effects upon repeated application. The bacterial alginate was not practically used for protein and peptide encapsulation for biomedical applications, especially for development of wound dressing with antibacterial activity. To confirm the suitability of bacterial alginate as a pharmacologically acceptable gelling agent, it must be shown that its origins, structure, and characteristics do not worsen its safety profile compared to traditional seaweed-derived alginate. In this regard, a targeted safety assessment of the developing gel appears to be a necessary step to justify its further preclinical and clinical development.

The absence of adverse effects, such as toxicity, inflammatory capacity, or immune reactions, is a critical step toward the effective application of medical materials. Evaluating cell viability and biocompatibility provides important information for *in vivo* development. During the study, we obtained gel compositions based on modified endolysin LysSi3-LK and capsular alginate (sodium alginate gel and calcium alginate hydrogel) produced by *Azotobacter vinelandii* by targeted biotechnological synthesis. Experiments studying the antibacterial activity of the gel showed its potential against multidrug-resistant strains and clinical isolates of Gram-negative ESKAPE bacteria. However, high concentrations of the polysaccharide matrix reduce the activity of the antibacterial component. The proliferation of HaCaT keratinocytes was stimulated by the hydrogel compositions, which is expected to have a positive effect on wound healing. At the same time, enzybiotics formulated with the sodium alginate and, to a lesser extent, hydrogel form revealed some cytotoxicity. They reduce the metabolic activity of skin cells by up to 70% at high concentrations and affect their mobility. Our findings suggest that bacterial alginate-based enzybiotics are promising candidates for treating skin injuries. They propose the preservation and controlled release of an active antibacterial component, as well as the biocompatibility of the material, to effectively address local infections.

## 2. Results

### 2.1. Preparation and Characterization of Bacterial Alginate-Based Compositions with LysSi3-LK

In this study, biosynthetic alginate, which was obtained by directed bacterial synthesis, was used as a matrix for encapsulating modified lysin and obtaining a prototype enzybiotic gel [32]. High-molecular-weight capsular alginate from *A. vinelandii* with the following parameters was selected: a molecular weight of 279 kDa, an M/G ratio of 55/45, and an acetylation level of 5.4% ± 1.4%. The gel compositions were prepared with bacterial alginate (bALG) concentrations of 1–4% and varying concentrations of the enzyme (0.1–1 mg/mL). As it was previously shown, the complex modulus G* of viscoelasticity of the bacterial alginate hydrogel is 1.1–1.4 kPa [32].

Two types of compositions were obtained during the course of the work: viscous and fluid gels based on sodium alginate salts and dense hydrogels based on calcium salts with a relatively rigid matrix structure. The former can be used as a standalone hydrophilic agent applied to wounds, burns, or trauma, while the latter can be used to develop alginate-based dressings for heavily exuding wounds, as scaffolds for tissue engineering and regenerative medicine, and as an antimicrobial component of dressings and covering materials. Hydrogel samples were made from alginate gels by cross-linking them through calcination. The gels had pronounced opalescence due to the formation of complexes between negatively charged alginate molecules and positively charged endolysin molecules.

**Figure 1:**
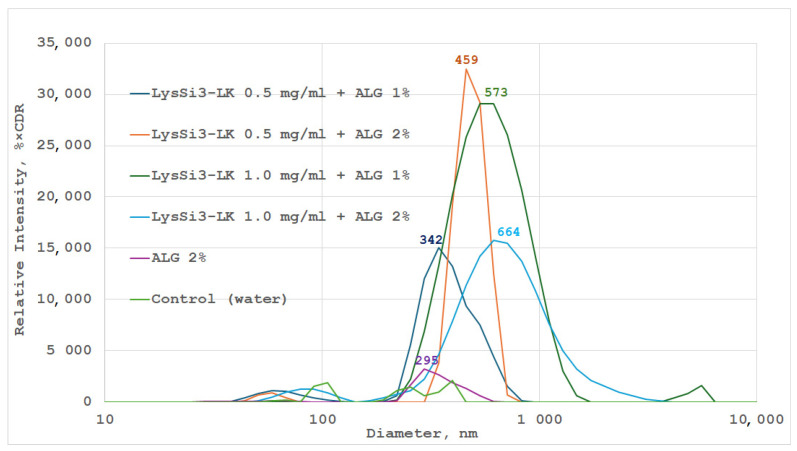
A comparison of the nanoparticle diameter in endolysin solutions at concentrations of 0.5 and 1 mg/mL with different concentrations of alginate (1% and 2%). DCR—Derived Count Rate.

Dynamic light scattering (DLS) studies of 1% and 2% alginate gels, containing 0.5–1.0 mg/mL of modified lysin, showed that the number of nanoparticles in the composition increases directly with the concentration of the modified lysin in alginate. The average particle size ranged from 342 to 664 nm (Figure 1), increasing with the concentrations of both the polymer and the enzyme. This confirms the formation of stable dispersions similar to those of specially prepared, stabilized emulsions from supramolecular protein-alginate complexes [33].

The average diameter of the polysaccharide-protein complexes formed in solution exceeds that of the nanoparticles formed in a pure bacterial alginate solution. Additionally, regardless of alginate concentrations, the polysaccharide-protein complexes have a relatively narrow size distribution of ±200–300 nm, indicating the formation of stable dispersions. An increase in protein concentration leads to wider peaks in the spectra, indicating a broader range of particle sizes. After the formation of hydrogels, a pronounced correlation was observed between an increase in endolysin content and a decrease in microsphere transparency, manifested by a progressive turbidity increase.

The most likely mechanism for complex formation is an electrostatic interaction between the anionic carboxyl groups of alginate and the cationic sites of endolysin. In this case, a single protein molecule can bind to multiple polysaccharide chains simultaneously, resulting in particle enlargement and increased light scattering intensity [34,35]. Additionally, the formation of a calcium alginate hydrogel matrix in the presence of Ca^2+^ ions may result in crosslinking heterogeneity and local Ca^2+^ concentration gradients [36]. In these conditions, protein-polysaccharide complexes can become fixed in the cross-linking matrix and aggregate with each other, while calcium ions partially neutralize the negative charge of alginate and contribute to the formation of protein-enriched domains. After the initial electrostatic interaction, hydrogen bonds and weak hydrophobic interactions may provide additional stabilization [37].

**Figure 2:**
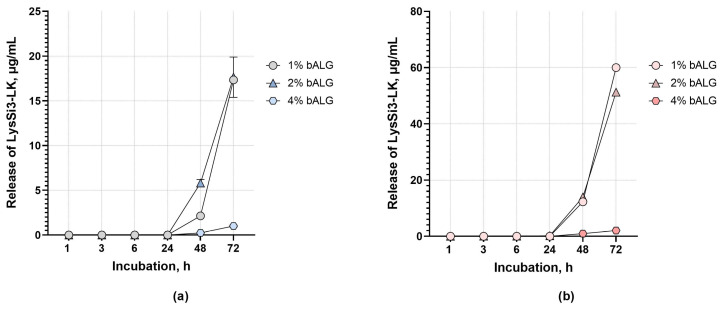
The dynamics of LysSi3-LK release from bacterial alginate-based gels at concentrations of (**a**) 0.1 mg/mL and (**b**) 0.5 mg/mL. Data are presented as mean values ± standard deviation (SD).

The kinetics of LysSi3-LK endolysin release from alginate matrices were studied. In all cases, endolysin release from the composition began after six hours of incubation, and active enzyme diffusion was observed after 24 h (Figure 2).

The release rate depended on the gel base concentration. The most rapid release was observed in the 1–2% gel base matrix, whereas the 4% alginate gel formulation exhibited low protein release, amounting to 1–2 μg/mL by the third day, regardless of the encapsulated enzyme concentration. This may be due to stronger electrostatic binding resulting from a greater number of positively charged carboxyl groups binding to the endolysin. Thus, gels with a high polymer content release enzyme more slowly. At the same time, at least 80% of the released enzyme activity was retained compared to the pure enzyme solution. Therefore, encapsulating lysin in an alginate matrix ensures the protein is gradually released in its active form in the obtained compositions.

### 2.2. Antibacterial Activity of Bacterial Alginate-Endolysin Gels

As previous studies have shown, the endolysin LysSi3-LK exhibits a broad spectrum of antibacterial activity against ESKAPE bacteria [38]. However, forming protein-polysaccharide complexes when the enzyme is incorporated into alginate gels can significantly impact its antibacterial activity.

**Figure 3:**
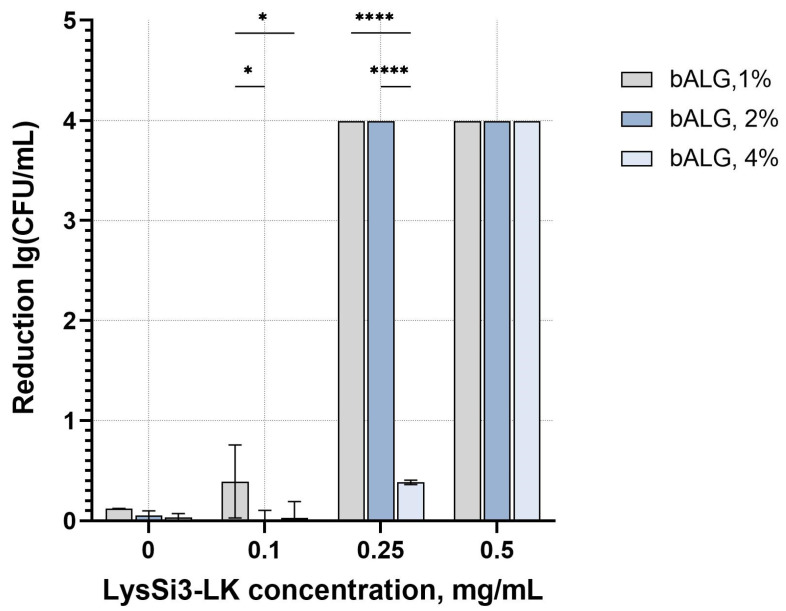
The dose-dependent activity of bALG-based gels against *A. baumannii* ATCC 19606 model strain. Data are presented as mean values ± SD; *—*p* < 0.05; ****—*p* < 0.0001; two-way ANOVA.

We evaluated the activity of LysSi3-LK at concentrations of 0.1, 0.25, and 0.5 mg/mL in gels with different concentrations of bacterial alginate on a model *A. baumannii* strain sensitive to LysSi3-LK action (Figure 3). The results showed that the activity of the composition depends on the content of both lysin and bALG. The bactericidal effect of the composition was observed starting at the enzyme concentration of 0.1 mg/mL at a sodium alginate concentration of 1%, but was absent in gels with higher polymer concentrations. For gels with a higher concentration of alginate (2% and 4%), the activity was observed at a concentration of the protein of 0.25 mg/mL, but was less pronounced in the more concentrated gel. The gel bases themselves exhibited no significant antibacterial activity, regardless of concentration.

The effect of lysin encapsulation on its spectrum of activity against the Gram-negative ESKAPE pathogens (*A. baumannii*, *Pseudomonas aeruginosa*, *Klebsiella pneumoniae* and *Escherichia coli*) was also evaluated. A comparison was made between the activity of non-encapsulated lysin at a concentration of 0.5 mg/mL and LysSi3-LK in the 2% gel (Table 1).

The antibacterial properties of the modified LysSi3-LK enzyme are affected by the encapsulation in the polymeric polysaccharide matrix of bacterial alginate. Activity against most strains remained high (over 70%), reaching 100% for *A. baumannii* and *P. aeruginosa*. However, the results for Enterobacteriaceae (*E. coli* and *K. pneumoniae*) were inconsistent and depended on the strain studied. The reasons for this selective activity decrease are not yet fully understood and require further research.

### 2.3. Evaluation of Cytotoxic Effects of LysSi3-LK Encapsulated in Bacterial Alginate Composition

Cytotoxicity testing is crucial for assessing the impact of new materials on cellular viability at the early stages of development. The cytotoxic effects of the sodium alginate-based formulation and its individual components were evaluated based on their ability to affect the metabolic activity of keratinocytes (the cells that form the basis of the skin’s epidermis) using the MTT assay. The evaluation started at concentrations of 2% bALG and 0.5 mg/mL LysSi3-LK.

**Figure 4:**
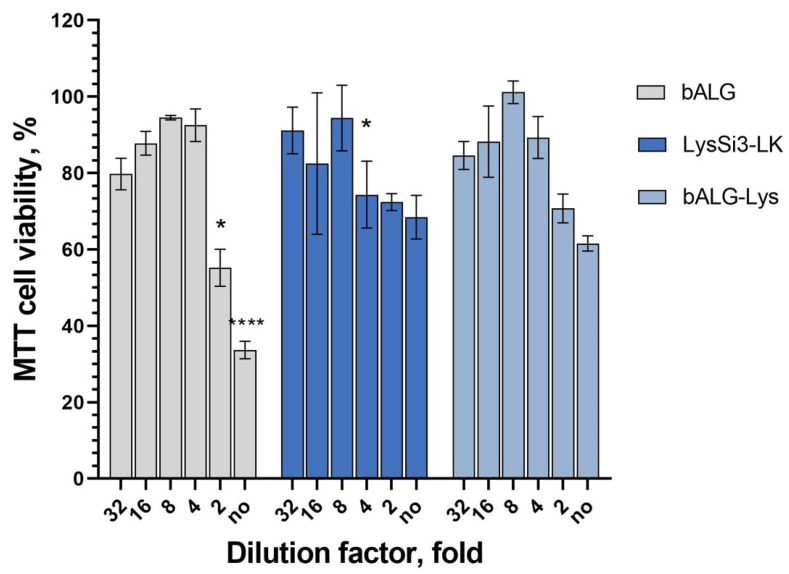
HaCaT cell viability in the presence of gel components and the composition. bALG, bacterial sodium alginate; bALG-Lys, composition of sodium alginate (2% initial concentration) and LysSi3-LK (0.5 mg/mL initial concentration). The mean values for three replicates are shown for all groups of samples (± SD). *—*p* < 0.05, ****—*p* < 0.0001 compared to bALG-Lys group, two-way ANOVA.

The impact of the lysin solution on HaCaT viability was negligible. This effect was most pronounced at protein concentrations above 0.25 mg/mL, where cell viability was at a 69–72% level. In contrast, *A. vinelandii* sodium alginate alone exhibited a more pronounced cytotoxic effect at concentrations above 1%, resulting in a reduction in the number of viable cells to 34–55% of the intact control (Figure 4).

At the same time, incorporating modified endolysin into the sodium alginate matrix significantly reduced the composition’s cytotoxicity compared to the bALG. At maximum alginate concentrations (1–2%) in the lysin-containing formulation, the percentage of viable cells increased to 62–70% (compared to 34–55% in the vehicle gel). The cytotoxicity of concentrated alginate can be explained by the impaired transport of oxygen, metabolites, and nutrients to cells through the viscous gel layer.

### 2.4. Assessment of the Compositions’ Effect on Cell Migration

**Figure 5:**
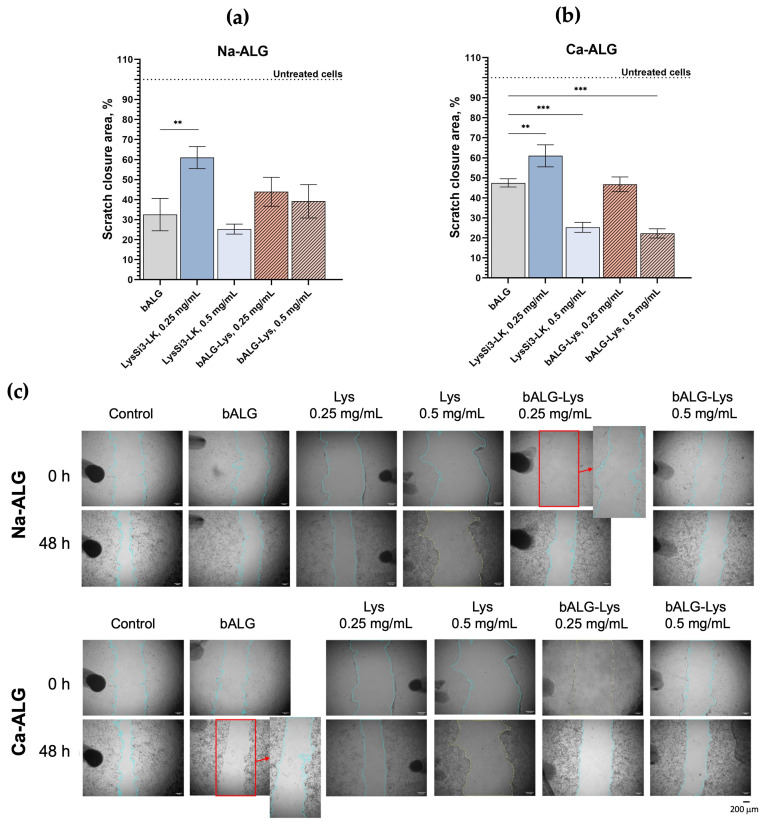
Results of the migration assay of HaCaT cells with the investigated preparations within 48 h. Scratch closure area, which was occupied by the cells for: (**a**) Bacterial sodium alginate gel samples; (**b**) Bacterial calcium alginate hydrogel samples. Data are presented as mean values ± SD. (**c**) Bright field light microscopy (scale bar, 200 μm). Na-ALG, bacterial sodium alginate gels; Ca-ALG, bacterial calcium alginate hydrogels; Lys, endolysin LysSi3-LK. **—*p* < 0.01, ***—*p* < 0.001 compared to bALG group, one-way ANOVA.

The migration rate of HaCaT cells was assessed using a scratch assay with sodium bALG gels and sphere-shaped calcium hydrogels at concentrations of the components that exhibited moderate cytotoxicity while retaining antibacterial activity (1% bALG and 0.25–0.5 mg/mL LysSi3-LK) (Figure 5).

All of the tested compounds (modified lysin, sodium alginate, calcium alginate, and compositions) significantly affected cell migration. Cells exhibited maximum migration in the presence of 0.25 mg/mL LysSi3-LK in both the free and alginate-encapsulated form (61% and 44–47% of the control rate, respectively). At a concentration of 0.5 mg/mL, the enzyme had a significantly greater impact on HaCaT migration, reducing it to 22–39% of the migration rate of intact cell layers. Also, the alginates themselves had a pronounced effect on the cells, leading to a loss of motility of up to 32.5% and 47.5% for Na-ALG and Ca-ALG, respectively. 

### 2.5. Evaluation of Biocompatibility of Endolysin LysSi3-LK Encapsulated in Alginate Films

**Figure 6:**
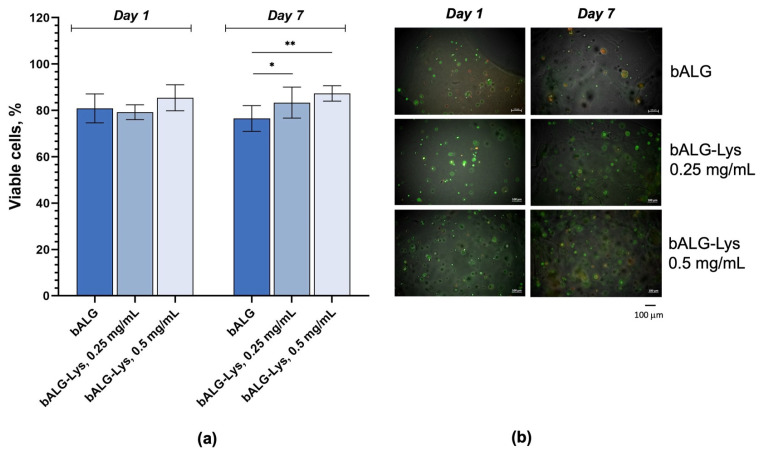
HaCaT cells viability dynamics in the biocompatibility assay: (**a**) Viable cells percent, mean values are presented ± SD; (**b**) Fluorescence microscopy of HaCaT incorporated into bALG calcium-alginate films with addition of the endolysin LysSi3-LK in 0.25 and 0.5 μg/mL concentrations. *—*p* < 0.05; **—*p* < 0.01, two-way ANOVA.

The viability and proliferation of HaCaT cells within 2% calcium alginate films containing 0.25 and 0.5 mg/mL endolysin were analyzed using a live/dead fluorescent staining assay. The results showed that the percentage of viable cells remained above 75% throughout the observation period in all the composites studied. The day after the compositions were added, the cells began to proliferate slightly in the presence of LysSi3-LK in a dose-dependent manner (Figure 6), and by day 7, this difference had become significant.

Hydrogels without an enzyme did not support significant cell growth; however, cell viability remained at a 76.5–81% level throughout the week of observation. For formulations containing lysin, an increase in enzyme concentration was accompanied by more pronounced cell proliferation, as evidenced by an increase in the number of live cells to 86–87% by day 7. A similar tendency was observed when cell adhesion agents (including protein-based agents, such as peptides) were incorporated into hydrogel matrices to enhance cell attachment. It was shown that composite alginate hydrogels without cell adhesion peptides did not support significant cell growth; however, cell viability was maintained after five days of incubation on the hydrogels [39]. Therefore, LysSi3-LK can also act as a cell adhesion agent, promoting cell attachment and growth.

## 3. Discussion

Alginates are biocompatible polymers that promote cell growth and proliferation [40]. These biodegradable polymers break down without producing toxic compounds. In therapeutic formulations, alginate can act as a substrate that facilitates cell migration, proliferation, and differentiation. It is essential that composite antimicrobial materials maintain active metabolism and the ability of cells to proliferate within the matrix so that they can be successfully used in treating wound infections. This ensures not only the antimicrobial effect of the therapeutic agent, but also its wound-healing properties. In this regard, bacterial alginates produced through directed synthesis under controlled biotechnological conditions could be a promising alternative to natural algal polymers. However, unlike well-studied algal alginates, bacterial polymers remain relatively understudied in terms of their potential toxic effects. Furthermore, differences in production methods and sources may affect their biocompatibility.

Although alginate-based materials are considered to be low- or non-toxic, some data suggest their potential side effects. Treatment of fibroblasts with extracts from bacterial alginate sheets within 72 h led to 85% cell viability, proving the principal biocompatibility of the bacterial alginate dressings [41]. At the same time, it has been demonstrated that alginates with increased M-block and MG-block contents can promote cytokine production [42]. A study on mesenchymal stem cells showed that bacterial alginate is less toxic than algal alginate [31]. However, it should be noted that free bacterial alginates with a low molecular weight (up to 100 kDa) have a greater cytotoxic effect than those with a higher molecular weight, as well as capsular alginates [32]. Furthermore, when the ability of algal and bacterial alginates to absorb various wound markers *in vitro*, such as proteases, pro-inflammatory cytokines, and reactive oxygen species, was compared, it was found that microbial alginate binds larger amounts of elastase and matrix metalloproteinase-2, indicating an increased potential to heal damaged wound surfaces [43].

The capacity of alginates to efficiently bind peptide and protein molecules is currently being investigated for its potential application in the development of enzybiotics for topical treatment. The potential for using algal polymers as a carrier matrix for antibacterial proteins has been assessed in several studies. Ghate et al. confirmed that binding to alginate stabilized the structure of T4L and T7L endolysins, thereby enhancing their antibacterial activity [44]. Subsequently, the pH-dependent release of endolysins from calcium alginate granules while preserving their lytic activity was investigated [45]. Another study revealed that a gel composed of algal alginate and containing a combination of two endolysins effectively eradicates mixed and monospecific biofilms while maintaining non-toxicity towards eukaryotic cells [4]. Encapsulation of the chimeric endolysin ClyC into an alginate hydrogel has been demonstrated to promote sustained release and maintain enzyme activity, consequently leading to a reduction in bacterial load in a murine model of osteomyelitis [19]. Therefore, preparations based on endolysins and alginates are regarded as a promising approach for combating resistant microorganisms that cause skin and soft tissue infections.

We previously obtained a recombinant modified endolysin LysSi3-LK, based on LysSi3 muramidase [38,46], which was hybridized with an amphiphilic alpha-helical peptide consisting of leucine and lysine residues. This hybrid molecule is capable of permeabilizing the outer membrane of Gram-negative bacteria and possesses activity against several ESKAPE species. In this study, we encapsulated the enzyme within a polysaccharide matrix of sodium and calcium alginate to evaluate the resulting compositions’ antibacterial and cytotoxic properties. The gels used in the study were formulated with bacterial capsular alginate, which has a molecular weight of 279 kDa and a M/G ratio of 55/45. The polymer concentration in the gels ranged from 1 to 4%, which has sufficient viscosity for application to the skin. The lysin concentration was 0.1–1 mg/mL, which corresponds to effective doses of the purified enzyme.

Encapsulating LysSi3-LK in bacterial alginate gel matrices formed stable dispersions of protein-polysaccharide (PS) complexes (nanoparticles or micelles), ensuring the sustained release of endolysin in its active form. A study of the kinetics of lysin release from alginate compositions revealed that LysSi3-LK was actively released in the free form after 24 h of incubation *in vitro*. However, the complexes also exhibited antibacterial activity. Gels with lower polysaccharide content showed the highest antibacterial activity when gels with different alginate contents (1%, 2%, and 4%) were compared. This correlates with the dynamics of enzyme release from the matrix and its viscosity. It may be explained by the fact that gels with lower ALG content—and consequently, lower viscosity—less actively form complexes with endolysin, whereas more concentrated and denser matrices limit enzyme diffusion. However, despite the low LysSi3-LK release rate from 4% sodium alginate compared to 1–2% gels, the concentrated formulation exhibits high activity at elevated protein concentrations (100% at 0.5 mg/mL). This indicates that the bound lysin retains its properties upon encapsulation. Thus, the formation of relatively large protein-PS complexes does not block the protein’s catalytic center or enzymatic functions.

Various aspects of the biological activity of the developed enzybiotics against human keratinocytes (HaCaT cell line) were examined, including the maintenance of active eukaryotic cell metabolism (MTT assay), their effect on cell migration, and the impact on proliferation within alginate films. HaCaT is a spontaneously immortalized human epidermal keratinocyte cell line that retains characteristics of differentiated cell layers of the human epidermis. It is one of the main cell types involved in the re-epithelialization process [47]. Thus, these cells were selected as a representative model in this study to assess the biocompatibility of the material for topical application.

Concentrations of bacterial sodium alginate above 1% were found to have a cytotoxic effect on keratinocytes. However, the addition of endolysin had a positive effect on cell survival. As previously mentioned, this observation can be explained by the viscosity of the matrix formed by high-molecular-weight alginate. A high-density environment significantly impedes the delivery of oxygen and nutrients to cells, affecting their survival. Adding protein to the matrix alters cell-gel interactions by changing the gel structure, degree of gel hydration, and screening of monomer charges. This potentially improves local transport and partially restores cellular metabolism [48,49]. Moreover, we hypothesize that structuring free-form linear alginate molecules into a nanoparticle dispersion with lysin improves nutrient access to cells.

Cell migration plays a key role in wound healing, and it was assessed for both gel components and compositions by creating a scratch in a confluent cell monolayer. Cell migration was significantly impaired under conditions that limited proliferative activity in the presence of the tested compositions. This effect was dose-dependent and more pronounced with calcium alginate spheres than with sodium alginate gels. This likely occurs due to the formation of a layer of the composition on the surface, which alters hydration and slows the rate of cell movement while maintaining viability. Both the alginate polymer and lysin are charged molecules that can bind to the surface, affecting keratinocyte adhesion and extracellular matrix surface density [50,51]. Previous studies have shown that higher concentrations of the alginate matrix can reduce cell adhesion, which can decrease viability in various cell lines [39,52,53]. In the case of the LysSi3-LK enzyme, the decrease in migration may be due to the sublethal effect of endolysins on eukaryotic cell membranes. The endolysin contains regions with cationic amino acids and a polycationic antimicrobial peptide that can interact with HaCaT lipid membranes [3,54]. While this might not be sufficient to cause cell death, it can disrupt membrane permeability or polarization, resulting in reduced motility. Thus, although mechanical forces appear to be the most obvious cause of the observed keratinocyte behavior in our study, the influence of the formulations and their components on chemotaxis and cellular signaling, which mediate cellular and physiological processes, cannot be completely discounted.

Our study shows that, in the presence of bacterial alginate compositions containing encapsulated modified lysin, keratinocytes maintain high levels of proliferation and metabolism (at least 75% of the control values), though their motility is reduced. Nevertheless, the observed pattern aligns with the generally accepted criterion of non-cytotoxicity and is consistent with data on the high biocompatibility of alginate hydrogels with cells [55]. 

To our knowledge, this is the first attempt to investigate the potential of bacterial alginates for enzybiotic development. We confirmed that bALG ensures the controlled release of the active form of endolysin and preserves enzyme activity within protein-PS complexes. It also maintains skin cell viability and stimulates the proliferation of cells within the hydrogel. This allows the substance to gradually be replaced by newly formed tissue and promotes wound healing in cases of damage to the upper layers of the skin. Given the absence of critical changes in cell survival and proliferation *in vitro* we do not suggest serious limitations of *in vivo* application of alginate-based enzybiotic containing LysSi3-LK in concentrations less than 0.5 mg/mL; however this needs to be confirmed experimentally. While these data enable us to evaluate the potential impact of the studied composition on skin cells, they cannot be directly extrapolated to living organisms, whose skin epidermis has a more intricate structure comprising various cell types. *In vivo* wound healing process is considerably more complex, necessitating the examination of the rate of wound healing in animals, along with the impact on skin cell composition and molecular markers.

## 4. Materials and Methods

### 4.1. LysSi3-LK Production and Purification

The modified LysSi3-LK enzyme sequence was created using a genetic construct based on a pET-42b(+) expression vector containing the LysSi3 endolysin sequence [46]. The additional LK peptide [56]-encoding insert was constructed from oligonucleotide primers using the overlapping fragment method.

The target fragments were amplified and analyzed using agarose gel electrophoresis. Then, the fragments were excised from the gel and purified using a QIAquick Gel Extraction Kit (Qiagen, Venlo, The Netherlands), following the manufacturer’s protocol. The concentration of the purified fragments was determined using a Qubit^®^ 4.0 fluorometer (Invitrogen, Carlsbad, CA, USA).

The purified PCR products were ligated using T4 DNA polymerase (Thermo Fisher Scientific, Waltham, MA, USA) according to the manufacturer’s instructions. After ligation, the reaction mixture was incubated with a DpnI enzyme (Thermo Fisher Scientific, Waltham, MA, USA) for eight to ten hours to remove the template DNA. The resulting DNA was introduced into chemically competent *E. coli* Top10 cells (Invitrogen, Carlsbad, CA, USA) using the heat shock method. The grown colonies were analyzed by PCR to detect the target endolysin sequence. Plasmid DNA was isolated using a Plasmid Miniprep kit (Evrogen, Moscow, Russia) according to the manufacturer’s instructions. Sanger sequencing using BigDye™ Terminator v3.1 (Applied Biosystems, Foster City, CA, USA) confirmed the correctness of the construct by analyzing the open reading frame of the plasmid containing the endolysin gene and peptide modification.

The recombinant protein was expressed in the *E. coli* Rosetta (DE3) strain (Novagen, Madison, WI, USA) in 2×YT medium containing kanamycin and chloramphenicol. Induction was performed with 1 mM isopropyl β-d-1-thiogalactopyranoside at an optical density of 0.6–0.8 o.u. in the bacterial culture, and afterwards, the cells were cultured for 3–4 h. Purification was performed using two-step chromatography of the soluble lysate fraction, with the protein first being separated using an SP-Sepharose cation exchange column (GE Healthcare, Chicago, IL, USA) and gel filtration then being performed using a Superdex 75 pg column (GE Healthcare, Chicago, IL, USA) in phosphate-buffered saline (PBS, pH 7.4; VWR, Radnor, PA, USA). The solution was dialyzed into the 20 mM Tris-HCl (pH 7.5) buffer solution. The protein concentration was determined spectrophotometrically using a theoretical absorption coefficient of 1.315 (Implen, München, Germany). The resulting preparation was lyophilized and stored at −30 °C until further experiments.

### 4.2. Bacterial Alginate Production

Previously, conditions were optimized for the targeted synthesis of alginates with different properties, such as molecular weight, as well as the synthesis of capsular versus free alginate, using the *A. vinelandii* 12 strain [32]. To maintain the bacterial culture, a solid Ashby nutrient medium was used with the following composition: K_2_HPO_4_ 0.2 g/L, MgSO_4_ 0.2 g/L, NaCl 0.2 g/L, Na_2_MoO_4_ 0.006 g/L, CaCO_3_ 5.0 g/L, sucrose 20.0 g/L, agar 20.0 g/L. Then, biosynthesis of alginate was performed by cultivation of the *A. vinelandii* 12 inoculum in 750 mL Erlenmeyer flasks containing 200 mL of Burke’s liquid medium with 35.0 g/L sucrose concentration. The cultivation process was conducted using a rotary shaker Innova 43 (New Brunswick Scientific, Edison, NJ, USA) at 250 rpm with the following parameters: initial medium pH of 7.2, cultivation temperature of 28 °C, process duration of 24 h, and inoculum volume of 4% (*v*/*v*).

After the fermentation process was completed, the biomass was separated from the culture broth by centrifugation at 11,000× *g* for 30 min. To isolate the capsular ALG, 45 mL of 1 M NaCl and 5 mL of 100 mM EDTA were added to the cell biomass. The mixture was then incubated for 1 h at 60 °C with continuous shaking using an orbital shaker PSU-20i (Bio-San, Riga, Latvia) to ensure complete homogenization. The supernatant was collected by centrifugation at 11,000× *g* for 30 min. Three volumes of cold ethanol were then added to the obtained supernatant, and the resulting precipitate was collected and lyophilized. Finally, dry ALG capsular precipitates were dissolved in 1 M NaCl and dialyzed against 1 L of 0.1 M NaCl for 30 h. After dialysis, the ALG was reprecipitated by adding three volumes of chilled ethanol, then lyophilized again. The molecular weight of the obtained ALG samples was determined by viscometry in an aqueous solution.

### 4.3. Preparation of Sodium Alginate Compositions

To sterilize the alginate powder of bacterial origin, a 0.5% sodium alginate solution was prepared by dissolving the powder in a 0.9% NaCl solution. Then, the solution was filtered through a 0.22-μm filter and mixed with 95% ethanol at a ratio of 1:3 to precipitate the alginate. After discarding the liquid, the alginate sediment was lyophilized overnight to obtain sterile, bALG powder.

The gel formulations were prepared aseptically, step by step, by adding a weighed portion of alginate to 20 mM Tris-HCl buffer (pH 7.5) to obtain base formulations with a twofold concentration of the gelling agent. The resulting alginate solutions were sterilized by autoclaving. Endolysin was subsequently added to the prepared gels and mixed for 1 h on a multi-rotator. The final concentrations of alginate and endolysin in the prepared sodium alginate solutions were: 1%, 2%, and 4% (*v*/*v*), both with and without the addition of LysSi3-LK at concentrations of 0.1, 0.25, and 0.5 mg/mL.

DLS was used to assess the efficiency of the gel formulation and the size of the particles formed. Measurements were performed using a Zetasizer Nano ZS instrument (Malvern Panalytical, Malvern, UK). Prior to analysis, the samples were centrifuged at 13,000× *g* for 10 min. Particle size distributions (nano- and microparticle fractions) were obtained from four replicate measurements using Zetasizer software (ver. 8.01.4906).

### 4.4. Preparation of Sphere-Shaped Calcium Alginate Compositions

**Figure 7:**
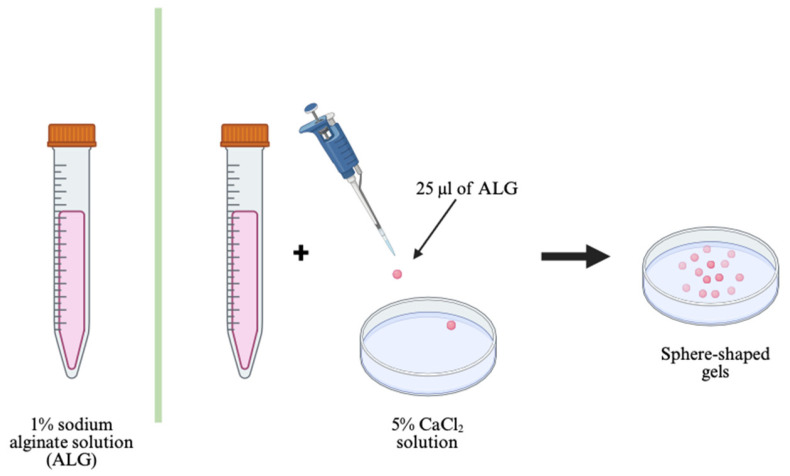
Scheme of sphere-shaped calcium alginate gels. Scheme was created in BioRender.com.

To prepare the sphere-shaped alginate gels, a 1% sodium alginate solution, with or without the addition of endolysins, was slowly added drop by drop to a 5% CaCl_2_ solution (Macklin, Shanghai, China). After the calcium alginate spheres formed, they were washed with DMEM (Biosharp, Beijing, China) and placed in plate wells (Figure 7).

The final amounts of alginate and endolysins in the prepared calcium alginate spheres were: 25 μL of 1% and 2% bacterial alginate, with and without the addition of endolysin LysSi3-LK, with final concentrations of 1000 μg/mL, 500 μg/mL, and 250 μg/mL.

### 4.5. Preparation of Calcium Alginate Films with Cells

To prepare calcium alginate films containing cells, a microscope glass slide was used as the base. Borders approximately 0.1 mm thick were fixed on both sides of the slide. A 4% sodium alginate solution was prepared by dissolving sterile bALG powder in water, as described in Section 4.3. This solution was then mixed with an endolysin solution and HaCaT cell suspensions to create a 2% sodium alginate solution with or without the endolysin LysSi3-LK and cells in a final concentration of 500,000 cells/mL.

**Figure 8:**
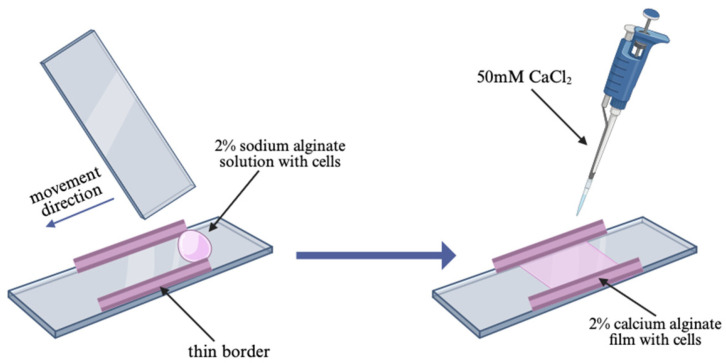
Scheme of calcium alginate films with cell preparation. Scheme was created in BioRender.com.

For each film, 150 µL of the sodium alginate solution containing cells and endolysins was placed on the slide between the borders. By moving another microscope slide, the solution was distributed equally between the two borders and covered with the solution for gelation (50 mM CaCl_2_ (Macklin, Shanghai, China), 10 mM HEPES (Macklin, Shanghai, China), and 0.9% NaCl (Macklin, Shanghai, China) (Figure 8). After 5–7 min, the film was washed with DMEM and cut into six equal pieces (approximately 0.5 cm^2^), which were placed in a 96-well plate. The final concentrations of endolysin LysSi3-LK in each well were 500 µg/mL and 250 µg/mL.

### 4.6. Study of LysSi3-LK Release from Alginate Compositions

A 200 µL aliquot of the gel sample was collected and transferred to an Amicon^®^ Ultra-0.5 ultrafiltration unit with a 100 kDa molecular weight cut-off (MWCO) (Merck KGaA, Darmstadt, Germany). The unit was then incubated in a thermoshaker at 250 rpm and 30 °C. The outer chamber of the Amicon device was filled with deionized water up to the level of the inner chamber containing the gel (1 mL), but not exceeding it. Release was assessed at the following time points: 1, 3, 6, 24, 48, and 72 h. At each time point, 100 µL was withdrawn from the outer chamber. This volume was then replenished with an equal amount of deionized water.

Quantification of endolysin in the gel formulation and in the release experiment was performed using an in-house ELISA test system. A 96-well plate was coated overnight at 4 °C with affinity-purified rabbit anti-LysSi3 antibodies (1.5 µg/mL) in carbonate–bicarbonate buffer (pH 9.3–9.6) for the sandwich ELISA. Then, the coating solution was removed, and the wells were blocked with S002 blocking solution (Xema, Moscow, Russia) for 1 h at room temperature. After blocking, the wells were emptied. Endolysin standards of known concentrations and test samples were diluted in ELISA diluent S011 (Xema, Moscow, Russia) and added to each well (100 µL) and incubated for 1 h at 37 °C. The wells were washed three times with 300 µL of wash buffer (PBS with 0.1% Tween-20). Then, 100 µL of anti-LysSi3 IgG-HRP conjugate (1:25,000 dilution) was added to each well and incubated for 1 h at 37 °C. After washing the wells five times, 100 µL of 3,3′,5,5′-tetramethylbenzidine (Xema, Moscow, Russia) substrate was added to each well. The wells were then incubated at room temperature for 10 min. The reaction was stopped with 10% HCl, and the absorbance was measured at 450 nm using a Multiscan FC plate reader (Thermo Fisher Scientific, Waltham, MA, USA). Endolysin concentrations were interpolated using the calibration curve obtained with serial dilutions of the LysSi3-LK standard solution.

### 4.7. In Vitro Assessment of Antibacterial Activity of Compositions Against Gram-Negative Bacteria

An overnight culture of *A. baumannii* ATCC 19606 strain in Mueller-Hinton broth (MHB; Himedia, Mumbai, India) was diluted in a fresh sterile medium and grown to the optical density OD_600_ of approximately 0.6–0.8 o.u. Then, the culture was centrifuged at 6000× *g* for 5 min and resuspended in sterile PBS to the turbidity, which was visually equal to the McFarland turbidity standard of 0.5. The suspensions were diluted 100-fold in a 20 mM Tris-HCl buffer at pH 7.5, resulting in a concentration of approximately 10^6^ CFU/mL. Then, 100 μL of tested gel samples were mixed with the prepared suspensions in a 96-well plate at a 1:1 ratio. 20 mM Tris-HCl buffer (pH 7.5) was used as a negative control. Then, the plate was incubated at 37 °C, 200 rpm for 1 h. Then, 10-fold dilutions of the mixtures were prepared in PBS and plated onto Petri dishes with Mueller-Hinton agar. The dishes were incubated at 37 °C overnight, after which the number of colony-forming units (CFUs) was assessed. The reduction in lg (CFU/mL) relative to the negative control was estimated. All experiments were carried out in triplicate.

The activity spectrum was studied by culturing and treating collection strains and clinical isolates of bacteria with the gel or its components, as described for *A. baumannii* ATCC 19606. Antibacterial activity was calculated using the following equation:

Antibacterial activity (%) = 100% − (CFUexp/CFUcont) × 100%,(1)

where CFUexp is the number of bacterial colonies in the experimental culture plates, and CFUcont is the number of bacterial colonies in the control culture plates.

### 4.8. Cytotoxicity Assessment of Compositions Using Keratinocytes 

The study involved sodium alginate-based formulations (see Section 4.3). HaCaT keratinocytes (ATCC PCS-200-011) were seeded into a 96-well plate for adherent cultures at a density of 20,000 cells/well. The cultures were incubated in 100 μL of full DMEM growth medium (DMEM supplemented with 10% fetal bovine serum) for 24 h at 37 °C and 5% CO_2_ in a humidified atmosphere until the monolayer reached 80–90% confluency. After incubation, 100 μL of two-fold serially diluted gel samples in DMEM were added to the plate. The concentrations of endolysin were 50, 25, and 12.5 μg/mL (from an initial concentration of 100 μg/mL in the gel), and 250, 125, 62.5, 31.25, and 15.63 μg/mL (from an initial concentration of 500 μg/mL). Samples of 4% diluted alginate gel without the addition of the enzyme were also prepared. 100 μL of DMEM medium was used as a negative control. For the positive control, 100 μL of a 0.1% Triton X-100 solution was added to the wells.

The plate was incubated at 37 °C with 5% CO_2_ for 24 h. Before staining, wells were washed with PBS to remove polymer materials. Then, 100 μL of MTT tetrazolium dye dissolved in DMEM medium at a concentration of 0.5 mg/mL was added to each well. The plate was then incubated for an additional 4 h at 37 °C and 5% CO_2_. Afterwards, the liquid was removed and 100 μL of DMSO was added to each well. The optical density (OD_570_) was measured using a microplate spectrophotometer SPECTROstar NANO (BMG LABTECH, Ortenberg, Germany). All experiments were carried out in triplicate.

The percentage of viable cells in each well was calculated using the following equation:

MTT cell viability (%) = (ODexp − ODmin)/(ODmax − ODmin) × 100%,(2)

where ODexp is the OD_570_ value in experimental wells, ODmax is the average OD_570_ value in DMEM medium wells (negative control), and ODmin is the average OD_570_ value in wells containing 0.1% Triton X-100 (positive control).

### 4.9. Cell Migration Assessment in the Presence of Alginate Compositions (Scratch Assay)

An *in vitro* scratch assay was performed to evaluate the effect of free and alginate-encapsulated endolysin LysSi3-LK on HaCaT cells’ migration and proliferation. For the experiment, LysSi3-LK solution and alginate compositions (see Section 4.3 and Section 4.4) with the endolysin in concentrations of 500 and 250 µg/mL were used.

HaCaT keratinocytes were seeded at a density of 30,000 cells/well and cultured in 100 µL of full medium supplemented with glutamate (DMEM; Biosharp, Beijing, China), 10% heat-inactivated FBS (Biosharp, Beijing, China), Antibiotic-Antimycotic (Gibco, Waltham, MA, USA), and GlutaMAX (Gibco, Waltham, MA, USA). The cells were then incubated at 37 °C in a humidified atmosphere containing 5% CO_2_ (Wiggens, Beijing, China) until they reached 80–90% confluence over 24 h. A linear scratch was created in each well using a sterile 200 µL pipette tip; the initial scratch width was approximately 600 µm. After forming the scratch, the wells were gently washed with PBS to remove detached cells and debris. Then, 100 µL of low-serum growth medium containing 1% FBS was added to each well, along with 25 µL of the samples. For samples containing endolysins, the low-serum growth medium did not contain Antibiotic-Antimycotic.

The following samples were tested for their effects on HaCaT cell migration and proliferation: 1% bacterial sodium alginate gels and 1% bacterial sphere-shaped hydrogels with and without the addition of 500 µg/mL and 250 µg/mL of endolysin LysSi3-LK; and free endolysin LysSi3-LK in concentrations of 500 µg/mL and 250 µg/mL. Each sample type was analyzed in three replicates. For the positive control, cells were cultured in 125 µL of the corresponding full growth medium.

Scratch closure was monitored using a bright field light microscope (Zeiss, Oberkochen, Germany) at 0, 24, and 48 h after scratch formation. Quantitative analysis was performed using ImageJ software (1.53t) (plugin: Wound_healing_size_tool_updated.ijm). For comparative analysis, the area covered by migrating cells in the positive control after 48 h was considered 100% migration. The area covered by migrating cells in the experimental groups was expressed as a percentage of the positive control.

### 4.10. Evaluation of Biocompatibility of Alginate Compositions on HaCaT Cells

To evaluate the biocompatibility of the endolysin LysSi3-LK encapsulated in Ca-ALG compositions, calcium alginate films containing HaCaT cells were prepared as previously described in Section 4.5. The final concentrations of endolysins in 2% bacterial alginate compositions in 96-well plates were 500 µg/mL and 250 µg/mL; films without endolysin were also prepared. The medium was changed on the 4th day of the experiment.

The proportion of viable and non-viable eukaryotic cells within the tested composition was evaluated using fluorescent staining with Sybr Green I (Thermo Fisher Scientific, Waltham, MA, USA) and propidium iodide (PI) (Solarbio, Beijing, China). The staining solution was prepared by diluting Sybr Green I (1000×) and PI (500×) in a 0.9% NaCl solution. Subsequently, 500 µL of the fluorescent staining mixture was added to each sample type in 2-mL centrifuge tubes. The staining procedure was conducted at 37 °C in a dark place for 20 min, followed by the washing of the samples with a 0.9% NaCl solution to ensure the removal of unbound dyes. 

Staining was conducted on days 1 and 7 from film preparation. The detection of Sybr Green I fluorescence was performed at λ = 522 nm, PI fluorescence—at λ = 618 nm.

The stained films were subsequently placed on microscope slides, and the ratio of number of living (Sybr Green I-positive) to dead (PI-positive) cells was determined using a fluorescence microscope Zeiss Axio Imager 2, (Zeiss, Oberkochen, Germany) by the following equation:

Viable cells (%) = (Living cells − Dead cells)/Living cells × 100%(3)

### 4.11. Summary of Formulation Tested During the Study

All samples used in the study are summarized in Table 2.

### 4.12. Statistical Analysis

All experiments were carried out in triplicate. Data are presented as mean ± SD. Statistical analysis was performed using ordinary one-way or two-way analysis of variance (ANOVA) with GraphPad Prism 9.5.0 software. Differences between experimental groups were considered statistically significant at *p* < 0.05.

## 5. Conclusions

The development of alginate-based gel and hydrogel formulations for the delivery of lytic enzymes is a promising strategy. Encapsulation of modified lysin LysSi3-LK allows the antibacterial activity of the enzyme to be preserved and ensures overall cytocompatibility. However, several limitations of the presented study should be acknowledged. On the one hand, the release model requires further optimization so it would better represent the changing conditions of wound infection, including pH shift, presence of proteases and other components of extracellular matrix. On the other hand, *in vitro* results demonstrating the reduction in cell viability at high concentrations and the inhibition of keratinocyte migration raise some concerns on regimens and safe doses of alginate-based enzybiotic that can be applied *in vivo* and in clinical practice. Thus, a more comprehensive characterization of skin irritation, immunological effects and repeated dose safety is still required. Safety experiments conducted on an animal model would provide more relevant conditions, better reflecting the pathophysiology of acute and chronic human wound infections under enzybiotic treatment.

## Figures and Tables

**Table 1:** The antimicrobial activity spectrum of the 2% bALG-based gel with lysin (0.5 mg/mL) and its individual components. The data are presented as mean values of the antibacterial activity ± SD.

Bacterial Strain	bALG, 2%	LysSi3-LK, 0.5 mg/mL	bALG-Lys
*A. baumannii* 50-16	3.02 ± 0.12	100 ± 0.00	100 ± 0.00
*A. baumannii* ATCC 19606	0 ± 7.48	100 ± 0.00	100 ± 0.00
*P. aeruginosa* ATCC 9027	16.21 ± 4.34	100 ± 0.00	100 ± 0.00
*P. aeruginosa* ATCC 10145	0 ± 7.00	100 ± 0.00	99.84 ± 0.05
*K. pneumoniae* 141-14	64.07 ± 12.21	100 ± 0.00	74.24 ± 4.14
*K. pneumoniae* ATCC 10031	22.16 ± 6.97	99.92 ± 0.00	94.86 ± 0.63
*E. coli* ATCC 259222	41.27 ± 3.19	99.72 ± 0.00	43.16 ± 1.31
*E. coli* ATCC 11229	3.35 ± 12.15	100 ± 0.00	81.99 ± 0.06

**Table 2:** Samples used in assays.

	Assay	Control Samples	Studied Samples
Concentrations of LysSi3-LK and bALG			
1	DLS	Water, bALG 2%	0.5 mg/mL + 1% 0.5 mg/mL + 2% 1.0 mg/mL + 1% 1.0 mg/mL + 2%
2	Release assay	-	0.1 mg/mL + 1% 0.1 mg/mL + 2% 0.1 mg/mL + 4% 0.5 mg/mL + 1% 0.5 mg/mL + 2% 0.5 mg/mL + 4%
3	Dose dependence	bALG 1% bALG 2% bALG 4%	0.1 mg/mL + 1% 0.1 mg/mL + 2% 0.1 mg/mL + 4% 0.25 mg/mL + 1% 0.25 mg/mL + 2% 0.25 mg/mL + 4% 0.5 mg/mL + 1% 0.5 mg/mL + 2% 0.5 mg/mL + 4%
4	Activity spectrum	bALG 2% Lys 0.5 mg/mL	0.5 mg/mL + 2%
5	Cytotoxicity assessment (MTT)	bALG 2% Lys 0.5 mg/mL	0.5 mg/mL + 2%
6	Cell migration assay	bALG 1% Lys 0.25 mg/mL Lys 0.5 mg/mL	0.25 mg/mL + 1% 0.5 mg/mL + 1%
7	Biocompatibility assay	bALG 2%	0.25 mg/mL + 2% 0.5 mg/mL + 2%

## Data Availability

The original contributions presented in this study are included in the article. Further inquiries can be directed to the corresponding author.

## Abbreviations

The following abbreviations are used in this manuscript:

bALG	Bacterial alginate
Na-ALG	Sodium alginate gel
Ca-ALG	Calcium alginate hydrogel
bALG-Lys	Bacterial alginate gel loaded with LysSi3-LK
Lys	Modified endolysin LysSi3-LK

## References

[1] Rahman M.U., Wang W., Sun Q., Shah J.A., Li C., Sun Y., Li Y., Zhang B., Chen W., Wang S. (2021). Endolysin, a Promising Solution against Antimicrobial Resistance. Antibiotics.

[2] Pastagia M., Schuch R., Fischetti V.A., Huang D.B. (2013). Lysins: The Arrival of Pathogen-Directed Anti-Infectives. J. Med. Microbiol..

[3] Carratalá J.V., Arís A., Garcia-Fruitós E., Ferrer-Miralles N. (2023). Design Strategies for Positively Charged Endolysins: Insights into Artilysin Development. Biotechnol. Adv..

[4] Vasina D.V., Antonova N.P., Shidlovskaya E.V., Kuznetsova N.A., Grishin A.V., Akoulina E.A., Trusova E.A., Lendel A.M., Mazunina E.P., Kozlova S.R. (2024). Alginate Gel Encapsulated with Enzybiotics Cocktail Is Effective against Multispecies Biofilms. Gels.

[5] Hamed Z.O., Awni A.A., Abdulamir A.S. (2023). Novel Recombinant Endolysin Ointment with Broad Antimicrobial Activity against Methicillin-Resistant Staphylococcus Aureus Isolated from Wounds and Burns. Arch. Microbiol..

[6] Li C., Nyaruaba R., Zhao X., Xue H., Li Y., Yang H., Wei H. (2022). Thermosensitive Hydrogel Wound Dressing Loaded with Bacteriophage Lysin LysP53. Viruses.

[7] Liu H., Wei X., Peng H., Yang Y., Hu Z., Rao Y., Wang Z., Dou J., Huang X., Hu Q. (2024). LysSYL-Loaded PH-Switchable Self-Assembling Peptide Hydrogels Promote Methicillin-Resistant Staphylococcus Aureus Elimination and Wound Healing. Adv. Mater..

[8] Mursalin M.H., Astley R., Coburn P.S., Bagaruka E., Hunt J.J., Fischetti V.A., Callegan M.C. (2023). Therapeutic Potential of Bacillus Phage Lysin PlyB in Ocular Infections. mSphere.

[9] Roach D.R., Donovan D.M. (2015). Antimicrobial Bacteriophage-Derived Proteins and Therapeutic Applications. Bacteriophage.

[10] Dawson R.M., Liu C.Q. (2011). Analogues of Peptide SMAP-29 with Comparable Antimicrobial Potency and Reduced Cytotoxicity. Int. J. Antimicrob. Agents.

[11] Antonova N.P., Vasina D.V., Grigoriev I.V., Usachev E.V., Aleshkin A.V., Vorobev A.M., Laishevtsev A.I., Kapustin A.V., Savinov V.A., Anurova M.N. (2024). Pharmacokinetics and Preclinical Safety Studies of Modified Endolysin-Based Gel for Topical Application. J. Pharm. Sci..

[12] Pastagia M., Euler C., Chahales P., Fuentes-Duculan J., Krueger J.G., Fischetti V.A. (2011). A Novel Chimeric Lysin Shows Superiority to Mupirocin for Skin Decolonization of Methicillin-Resistant and -Sensitive Staphylococcus Aureus Strains. Antimicrob. Agents Chemother..

[13] Totté J.E.E., van Doorn M.B., Pasmans S.G.M.A. (2017). Successful Treatment of Chronic Staphylococcus Aureus-Related Dermatoses with the Topical Endolysin Staphefekt SA.100: A Report of 3 Cases. Case Rep. Dermatol..

[14] de Wit J., Totté J.E.E., van Mierlo M.M.F., van Veldhuizen J., van Doorn M.B.A., Schuren F.H.J., Willemsen S.P., Pardo L.M., Pasmans S.G.M.A. (2019). Endolysin Treatment against Staphylococcus Aureus in Adults with Atopic Dermatitis: A Randomized Controlled Trial. J. Allergy Clin. Immunol..

[15] Thapa R.K., Grønlien K.G., Tønnesen H.H. (2021). Protein-Based Systems for Topical Antibacterial Therapy. Front. Med. Technol..

[16] Gondil V.S., Chhibber S. (2021). Bacteriophage and Endolysin Encapsulation Systems: A Promising Strategy to Improve Therapeutic Outcomes. Front. Pharmacol..

[17] Silva M.D., Ray K., Gama M., Remenschneider A.K., Sillankorva S. (2022). Ex Vivo Transtympanic Permeation of the Liposome Encapsulated S. Pneumoniae Endolysin MSlys. Int. J. Pharm..

[18] Gondil V.S., Harjai K., Chhibber S. (2021). Investigating the Potential of Endolysin Loaded Chitosan Nanoparticles in the Treatment of Pneumococcal Pneumonia. J. Drug Deliv. Sci. Technol..

[19] Yao F., Wu X., Liao Y., Yan Q., Li Y. (2021). Smart Chimeric Lysin ClyC Loaded Alginate Hydrogel Reduces Staphylococcus Aureus Induced Bone Infection. Front. Mater..

[20] Ozdil D., Aydin H.M. (2014). Polymers for Medical and Tissue Engineering Applications. J. Chem. Technol. Biotechnol..

[21] Abdulsalam L., Abubakar S., Permatasari I., Lawal A.A., Uddin S., Ullah S., Ahmad I. (2025). Advanced Biocompatible and Biodegradable Polymers: A Review of Functionalization, Smart Systems, and Sustainable Applications. Polymers.

[22] Ren Y., Wang Q., Xu W., Yang M., Guo W., He S., Liu W. (2024). Alginate-Based Hydrogels Mediated Biomedical Applications: A Review. Int. J. Biol. Macromol..

[23] Angra V., Sehgal R., Kaur M., Gupta R. (2021). Commercialization of Bionanocomposites. Bionanocomposites Tissue Eng. Regen. Med..

[24] Gombotz W.R., Wee S.F. (1998). Protein Release from Alginate Matrices. Adv. Drug Deliv. Rev..

[25] Sun J., Tan H. (2013). Alginate-Based Biomaterials for Regenerative Medicine Applications. Materials.

[26] Zhang Y., Wei W., Lv P., Wang L., Ma G. (2011). Preparation and Evaluation of Alginate-Chitosan Microspheres for Oral Delivery of Insulin. Eur. J. Pharm. Biopharm..

[27] Abourehab M.A.S., Rajendran R.R., Singh A., Pramanik S., Shrivastav P., Ansari M.J., Manne R., Amaral L.S., Deepak A. (2022). Alginate as a Promising Biopolymer in Drug Delivery and Wound Healing: A Review of the State-of-the-Art. Int. J. Mol. Sci..

[28] Hay I.D., Rehman Z.U., Moradali M.F., Wang Y., Rehm B.H.A. (2013). Microbial Alginate Production, Modification and Its Applications. Microb. Biotechnol..

[29] Schandl S., Osondu-Chuka G., Guagliano G., Perak S., Petrini P., Briatico-Vangosa F., Reimhult E., Guillaume O. (2025). Acetylation of Alginate Enables the Production of Inks That Mimic the Chemical Properties of P. Aeruginosa Biofilm. J. Mater. Chem. B.

[30] Bonartseva G.A., Akulina E.A., Myshkina V.L., Voinova V.V., Makhina T.K., Bonartsev A.P. (2017). Alginate Biosynthesis by Azotobacter Bacteria. Appl. Biochem. Microbiol..

[31] Akoulina E., Dudun A., Bonartsev A., Bonartseva G., Voinova V. (2019). Effect of Bacterial Alginate on Growth of Mesenchymal Stem Cells. Int. J. Polym. Mater. Polym. Biomater..

[32] Dudun A.A., Akoulina E.A., Zhuikov V.A., Makhina T.K., Voinova V.V., Belishev N.V., Khaydapova D.D., Shaitan K.V., Bonartseva G.A., Bonartsev A.P. (2021). Competitive Biosynthesis of Bacterial Alginate Using Azotobacter Vinelandii 12 for Tissue Engineering Applications. Polymers.

[33] Nooshkam M., Varidi M., Zareie Z., Alkobeisi F. (2023). Behavior of Protein-Polysaccharide Conjugate-Stabilized Food Emulsions under Various Destabilization Conditions. Food Chem. X.

[34] Amara C.B., Eghbal N., Oulahal N., Degraeve P., Gharsallaoui A. (2016). Properties of Lysozyme/Sodium Alginate Complexes for the Development of Antimicrobial Films. Food Res. Int..

[35] Fuenzalida J.P., Nareddy P.K., Moreno-Villoslada I., Moerschbacher B.M., Swamy M.J., Pan S., Ostermeier M., Goycoolea F.M. (2016). On the Role of Alginate Structure in Complexing with Lysozyme and Application for Enzyme Delivery. Food Hydrocoll..

[36] Girón-Hernández J., Gentile P., Benlloch-Tinoco M. (2021). Impact of Heterogeneously Crosslinked Calcium Alginate Networks on the Encapsulation of β-Carotene-Loaded Beads. Carbohydr. Polym..

[37] Zhao N., Zou H., Sun S., Yu C. (2020). The Interaction between Sodium Alginate and Myofibrillar Proteins: The Rheological and Emulsifying Properties of Their Mixture. Int. J. Biol. Macromol..

[38] Klimova A., Dudun A., Grigoriev I., Antonova N., Akoulina E., Voinova V., Bonartsev A., Vasina D. (2025). Engineering of novel alginate-based gels with modified endolysin and bacterial alginates with enhanced antibacterial activity against ESKAPE pathogens, Posters. FEBS Open Bio.

[39] Hwang P.A., Huang P.S., Hsu F.Y. (2025). Development and Biocompatibility Assessment of Alginate–Ulvan Hydrogels for Potential Medical Use. Carbohydr. Polym. Technol. Appl..

[40] Varaprasad K., Jayaramudu T., Kanikireddy V., Toro C., Sadiku E.R. (2020). Alginate-Based Composite Materials for Wound Dressing Application:A Mini Review. Carbohydr. Polym..

[41] Hoefer D., Schnepf J.K., Hammer T.R., Fischer M., Marquardt C. (2015). Biotechnologically Produced Microbial Alginate Dressings Show Enhanced Gel Forming Capacity Compared to Commercial Alginate Dressings of Marine Origin. J. Mater. Sci. Mater. Med..

[42] Otterlei M., Østgaard K., Skjåk-Bræk G., Smidsr⊘d O., Soon-Shiong P., Espevik T. (1991). Induction of Cytokine Production from Human Monocytes Stimulated with Alginate. J. Immunother..

[43] Fischer M., Gebhard F., Hammer T., Zurek C., Meurer G., Marquardt C., Hoefer D. (2017). Microbial Alginate Dressings Show Improved Binding Capacity for Pathophysiological Factors in Chronic Wounds Compared to Commercial Alginate Dressings of Marine Origin. J. Biomater. Appl..

[44] Ghate M.M., Gulati K., Poluri K.M. (2024). Alginate Binding Enhances the Structural Stability and Potentiates the Lytic Activity of Bacteriophage Endolysin’s Partially Folded Conformation. Arch. Biochem. Biophys..

[45] Ghate M.M., Poluri K.M. (2025). In-Vitro Evaluation of Alginate as an Encapsulation Matrix for PH-Dependent Delivery of Bacteriophage T4L and T7L Endolysins. Int. J. Biol. Macromol..

[46] Antonova N.P., Grigoriev I.V., Lendel A.M., Usacheva O.V., Klimova A.A., Usachev E.V., Gushchin V.A., Vasina D.V. (2024). Engineering of Recombinant Endolysin LysSi3 to Increase Its Antibacterial Properties. Appl. Biochem. Microbiol..

[47] Wang J., He J., Zhu M., Han Y., Yang R., Liu H., Xu X., Chen X. (2022). Cellular Heterogeneity and Plasticity of Skin Epithelial Cells in Wound Healing and Tumorigenesis. Stem Cell Rev. Rep..

[48] Golmohamadi M., Wilkinson K.J. (2013). Diffusion of Ions in a Calcium Alginate Hydrogel-Structure Is the Primary Factor Controlling Diffusion. Carbohydr. Polym..

[49] Mavris S.M., Hansen L.M. (2021). Optimization of Oxygen Delivery Within Hydrogels. J. Biomech. Eng..

[50] Li S., Guan J.L., Chien S. (2005). Biochemistry and Biomechanics of Cell Motility. Annu. Rev. Biomed. Eng..

[51] Vakeri A., Boire A., Davy J., Hamon P., Bouchoux A., Bouhallab S., Renard D. (2024). Coacervation and Aggregation in Lysozyme/Alginate Mixtures. Food Hydrocoll..

[52] Lee K.Y., Mooney D.J. (2012). Alginate: Properties and Biomedical Applications. Prog. Polym. Sci..

[53] Serafin A., Culebras M., Collins M.N. (2023). Synthesis and Evaluation of Alginate, Gelatin, and Hyaluronic Acid Hybrid Hydrogels for Tissue Engineering Applications. Int. J. Biol. Macromol..

[54] Ma X., Wang Q., Ren K., Xu T., Zhang Z., Xu M., Rao Z., Zhang X. (2024). A Review of Antimicrobial Peptides: Structure, Mechanism of Action, and Molecular Optimization Strategies. Fermentation.

[55] Bohari S.P.M., Hukins D.W.L., Grover L.M. (2011). Effect of Calcium Alginate Concentration on Viability and Proliferation of Encapsulated Fibroblasts. Biomed. Mater. Eng..

[56] Kang S.J., Won H.S., Choi W.S., Lee B.J. (2009). De Novo Generation of Antimicrobial LK Peptides with a Single Tryptophan at the Critical Amphipathic Interface. J. Pept. Sci..

